# Emergence of *Mycobacterium orygis*: novel insights into zoonotic reservoirs and genomic epidemiology

**DOI:** 10.3389/fpubh.2025.1568194

**Published:** 2025-03-19

**Authors:** Benedict T. Hugh, Eby M. Sim, Taryn Crighton, Vitali Sintchenko

**Affiliations:** ^1^School of Medical Sciences, Faculty of Medicine and Health, University of Sydney, Darlington, NSW, Australia; ^2^The Centre for Infectious Diseases and Microbiology – Public Health (CIDM-PH), Westmead Hospital, Westmead, NSW, Australia; ^3^New South Wales Mycobacterium Reference Laboratory, Centre for Infectious Diseases and Microbiology Laboratory Services, NSW Health Pathology, Institute of Clinical Pathology and Medical Research, Westmead, NSW, Australia

**Keywords:** *Mycobacterium orygis*, tuberculosis, zoonotic infections, genomics, epidemiology

## Abstract

Tuberculosis (TB), caused by members of the *Mycobacterium tuberculosis* complex (MTBC), is a significant global health threat, with millions of cases diagnosed annually and an annual death toll exceeding 1.6 million. Zoonotic TB (zTB), transmitted between animals and humans, remains poorly understood and difficult to control. This narrative review examined current evidence of the emergence and transmission pathways of *Mycobacterium orygis*, a recently defined member of MTBC. The structured searches for published literature and genome sequence with relevant metadata were conducted using NCBI PubMed and GenBank, respectively. Population diversity was investigated using phylogenomic analysis. Despite significant gaps in current laboratory diagnostic capacity for TB, *M. orygis* has been documented in 14 countries from 5 continents across 17 host species. Many cases (≈40%) were diagnosed around The Subcontinent and associated with a diverse range of mammalian hosts. In India, zTB due to *M. orygis* appeared to be more prevalent than disease associated with *M. bovis* (another zoonotic member of the MTBC). The whole genome sequencing of *M. orygis* isolates highlighted high diversity associated with different ecological niches. The increasing world-wide prevalence of *M. orygis*, especially in Asia, highlighted its emergence as a significant pathogen with zoophilic and anthropophilic potential. The reviewed evidence suggested multiple transmission pathways between humans and domesticated and wild mammalian hosts. Enhanced TB laboratory diagnostics and surveillance are imperative for mitigating the spread of zTB including one caused by *M. orygis* in areas of established and currently unrecognized endemicity.

## Introduction

Tuberculosis (TB) is an ancient disease that kills more people every year than any other infectious disease (1, 2). There are approximately 11 million cases globally, resulting in up to 1.6 million deaths annually (3). In 2024 the World Health Organization (WHO) reported a global reduction in the number of people diagnosed with TB and receiving treatment in the year prior. However, this is only a slight reversal in the treatment disruptions caused by the COVID-19 pandemic (4). TB disproportionally affects people in low-income and middle-income countries (LMICs). South Asia (SA), which includes India, Nepal, Pakistan, Bangladesh, and Sri Lanka, accounts for almost 40% of global cases (5). TB thrives in impoverished regions where access to healthcare services is limited and social determinants of poor outcomes such as overcrowding and poor ventilation are common (6). There is a direct association between poverty and higher rates of TB, especially in ethnic minorities (7).

Tuberculosis is caused by genetically related members of the *Mycobacterium tuberculosis* complex (MTBC), which includes both animal- and human-adapted (*M. tuberculosis sensu stricto*) lineages. The MTBC consists of over 10 species of mycobacteria with highly conserved genomes (8, 9). In addition, each of these species occupy a distinct ecological niche and infect a range of host species (10). Globally, of the eight members of the MTBC that cause TB in humans, the primary causative agent is *M. tuberculosis*. However, seven other species responsible for both human and zoonotic infections have been identified, including *M. orygis, M. bovis, M. caprae, M. canettii, M. africanum, M. pinnipedii,* and *M. microti* (11). Zoonotic tuberculosis (zTB) has been previously defined as a human infection caused by *M. bovis*; however, it is now understood zTB can be a manifestation of TB in humans, caused by an animal adapted lineage and acquired from infected animals (12). Urbanization and the destruction of natural wildlife habitats have increased the risk of zTB and approximately one-third of all zoonotic cases originate from Asia (13). The genetic similarity between the MTBC species demands high-resolution diagnostic methods to accurately identify the causative agent, as well as to decipher transmission dynamics and drug resistance in implicated pathogens. However, timely recognition of zTB remains challenging and zTB has become a significant public health concern globally with the potential to disrupt production and trade of animal products for food and other applications. This review described the emerging evidence of the global spread and diversity in *M. orygis* genomes, affected hosts and pathways of transmission.

## Oryx bacillus and the subsequent reclassification to *Mycobacterium orygis*

**Figure 1:**
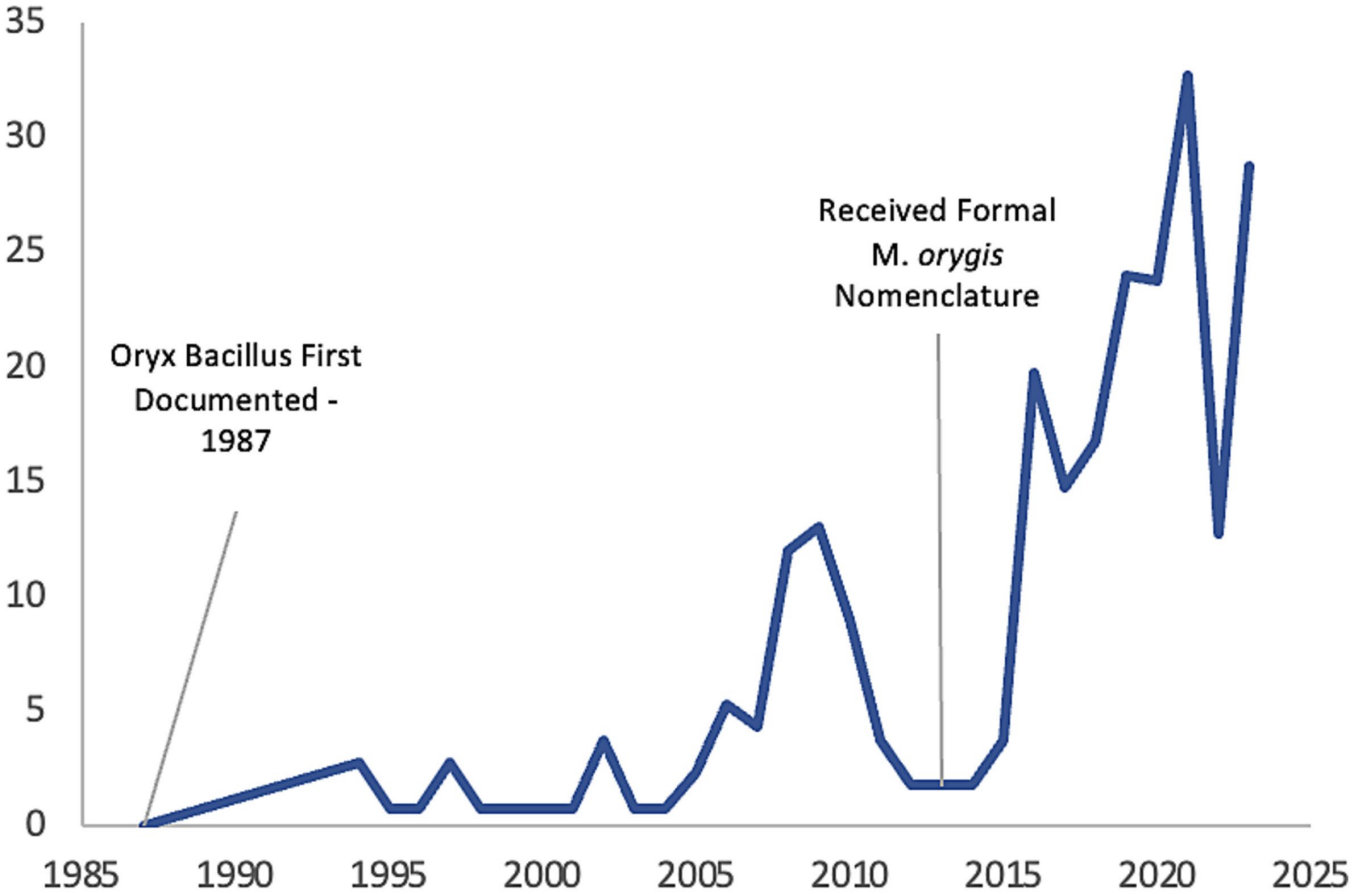
A historical timeline of *M. orygis* case reports.

Previously known as Oryx bacillus or the antelope clade, *M. orygis* has been infrequently reported in association with zTB over the past 30 years, although the number of case reports has recently increased due to advances in molecular diagnostics (Figure 1). In 1976, Lomme et al. observed granulomatous lesions in oryx’s that was suspected to be infected with *M. tuberculosis*; however, the oryx failed skin antigen tests and TB was confirmed by microscopic and postmortem examination (14). It has been subsequently reported that it was possible to have a negative tuberculous skin test (TST) in *M. orygis* positive cases (15). The first confirmed case of Oryx bacillus was diagnosed in 1987 by Van Soolingen et al. but was not reported until 1994 (16). Molecular typing using DNA fingerprinting revealed IS*6110* differences between *M. bovis* isolates and two *Oryx leucoryx* (Arabian oryx) isolates collected from the wildlife parks in the Netherlands and one isolate from a previous study (16). It should also be noted that methods for differentiation between members of the MTBC was in its infancy during that time. Formal nomenclature of *M. orygis* was proposed in 2012 following both molecular and genomic analyses demonstrating that Oryx bacillus, while genetically related to other members of MTBC, also possessed distinct genomic signatures which satisfied the definition for a new subspecies (17). Since then, reported case numbers have gradually increased, particularly over the past 10 years (Figure 1) (18).

## Global prevalence of *Mycobacterium orygis*

**Figure 2:**
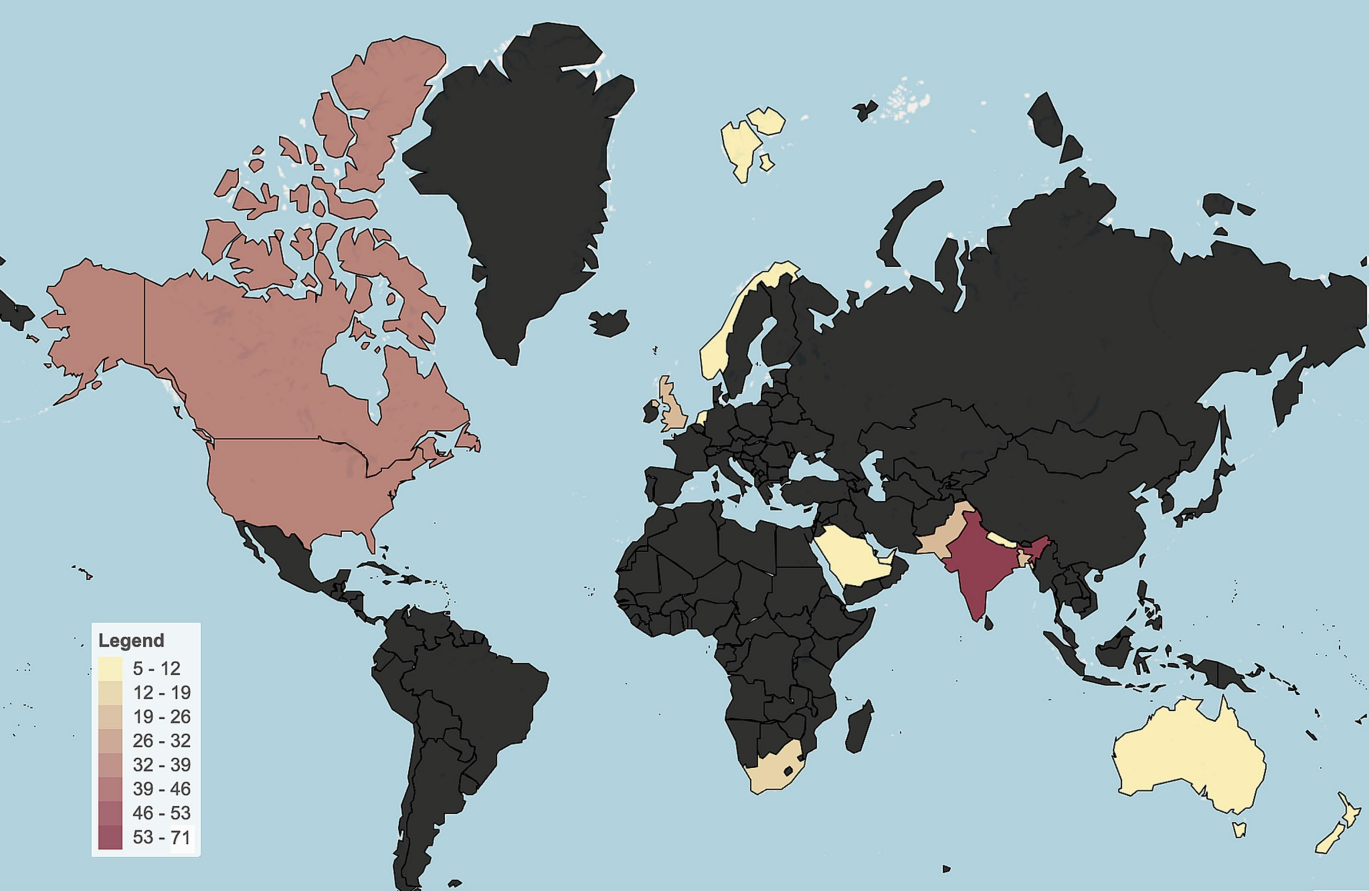
Global heat map of human and animal *M. orygis* infections reported between 1987 and 2024 using the data consolidated in Table 1. Cases are scaled according to the gradient bar and regions in gray indicate countries that have not yet reported any *M. orygis* infections.

As of March 2024, *M. orygis* was reported in 14 countries with 127 human and 123 animal infections across 17 unique host species (Table 1 and Figure 2). The only continents where the organism has yet to be reported are South America and Antarctica. A significant proportion of cases (84/250 or 33.6%) have been reported from India, Pakistan, Nepal, and Bangladesh. Importantly, these cases are associated with a diverse host range, spanning multiple mammalian species (19, 20). In the Subcontinent, the identification and reporting of *M. orygis* cases was primarily done through prospective studies (21). At the same time, in non-endemic countries, retrospective analysis of archived samples accounted for a more significant proportion of reported cases (Table 1) Identifying the MTBC subspecies is often not a priority or is unavailable, leading to gaps in epidemiological data and unrecognized transmission pathways (22).

**Table 1:** Summary of published *M. orygis* cases.

Country of diagnosis	Country of origin	Gender^#^	Host	Family	No. of cases	Sources of *M. orygis* culture^*^	References
Australia	India	1M, 7F	Human	Hominidae	8	N/A	(55)
Bangladesh	Bangladesh	N/A	Cattle	Bovidae	18	17 PTB, 1 EPTB	(19)
Bangladesh	Bangladesh	N/A	Rhesus Monkey	Cercopithecoidea	2	2 PTB	(19)
Canada	N/A	N/A	Human	Hominidae	1	EPTB (CNS)	(27)
Canada	N/A	14M, 26F, 2N/A	Human	Hominidae	40	26 PTB, 14 EPTB	(8)
Canada	India (18) & Pakistan (3)	2M, 19F	Human	Hominidae	21	10 PTB, 5 EPTB, 6 PTB & EPTB	(30)
India	India	N/A	Cattle	Bovidae	1	PTB	(56)
India	India	2M	Blackbuck	Bovidae	2	2 PTB & EPTB	(20)
India	India	N/A	Cattle	Bovidae	4	4 PTB & EPTB	(57)
India	India	N/A	Buffalo	Bovidae	1	PTB & EPTB	(57)
India	India	N/A	Sambar (Deer)	Cervidae	4	4 PTB & EPTB	(57)
India	India	N/A	Cattle & Buffalo	Bovidae	8	6 PTB & 2 EPTB	(58)
India	India	3M, 5F	Human	Hominidae	8	4 EPTB & 5 PTB	(45)
India	India	1M	Bison	Bovidae	1	PTB, EPTB	(59)
Netherlands^[a]^	South Asia	N/A	Human	Hominidae	9	4 EPTB & 5 PTB	(17)
India	India	N/A	Human	Hominidae	7	1 PTB, 6 EPTB	(28)
India	India	N/A	Cattle	Bovidae	4	4 PTB & EPTB (liver, mesenteric lymph nodes)	(60)
India	India	N/A	Buffalo	Bovidae	3	2 PTB, 1 PTB & EPTB (mesenteric lymph nodes)	(60)
India	India	N/A	Spotted deer	Cervidae	2	2 PTB	(60)
India	India	1M, 1F	Spotted deer	Cervidae	2	2 PTB & EPTB	(59)
India	India	1F	Spotted deer	Cervidae	1	PTB & EPTB	(20)
Nepal	Nepal	1F	Greater One Horned Rhinoceros	Rhinocerotidae	1	PTB & EPTB	(21)
Nepal	Nepal	N/A	Blue Bull	Bovidae	1	PTB	(61)
Nepal	Nepal	N/A	Spotted deer	Cervidae	1	PTB & EPTB	(61)
Netherlands^a^	Netherlands	N/A	Unspecified Animal	N/A	1	N/A	(17)
Netherlands	Saudi Arabi	N/A	Oryx	Bovidae	1	N/A	(16)
Netherlands	N/A	N/A	Deer	Cervidae	1	N/A	(48)
Netherlands	N/A	N/A	Waterbuck	Bovidae	1	N/A	(48)
Netherlands	N/A	N/A	Gazelle	Bovidae	1	N/A	(48)
Netherlands	Saudi Arabia	N/A	Oryx	Bovidae	2	N/A	(62)
Netherlands	N/A	N/A	Cattle	Bovidae	3	N/A	(16)
Netherlands	N/A	N/A	Oryx	Bovidae	1	N/A	(63)
Netherlands^a^	Netherlands	N/A	Waterbuck	Bovidae	3	N/A	(17)
Netherlands^a^	Netherlands	N/A	Antelope	Bovidae	1	N/A	(17)
Netherlands^a^	Netherlands	N/A	Oryx	Bovidae	1	N/A	(63)
Norway	South Asia	N/A	Human	Hominidae	5	3 PTB & 2 EPTB	(64)
New Zealand	N/A	1F	Cattle	Bovidae	1	EPTB	(27)
New Zealand	India	1F	Human	Hominidae	1	PTB	(27)
Pakistan	Pakistan	1F	Cattle	Bovidae	1	PTB & EPTB	(65)
Pakistan	Pakistan	1M, 8F	Buffalo	Bovidae	9	5PTB, 4 PTB & EPTB	(65)
Saudi Arabia	Saudi Arabia	N/A	Oryx	Bovidae	1	N/A	(16)
South Africa	N/A	N/A	Buffalo	Bovidae	7	4 PTB & 3 EPTB	(66)
South Africa	Portugal	1F	African Buffalo	Bovidae	1	PTB	(32)
South Africa	N/A	N/A	Oryx	Bovidae	1	N/A	(67)
Netherlands^a^	South Africa	N/A	Antelope	Bovidae	1	N/A	(16)
Netherlands^a^	Southeast Asia	N/A	Human	Hominidae	1	PTB	(16)
Netherlands^a^	Bangladesh	N/A	Cow	Bovidae	2	N/A	(16)
Netherlands^a^	Bangladesh	N/A	Monkey	Cercopithecoidea	1	N/A	(16)
United Arab Emirates	N/A	1M	Camel	Camelidae	1	PTB	(68)
United Arab Emirates	N/A	N/A	Camel	Camelidae	2	PTB & EPTB	(69)
Netherlands^a^	UK	N/A	Deer	Cervidae	1	N/A	(17)
UK	N/A	N/A	Human	Hominidae	24	N/A	(31)
USA	Africa	2F	East African Oryx	Bovidae	2	PTB & EPTB	(14)
USA	India	1M	Greater One Horned Rhinoceros	Rhinocerotidae	1	PTB & EPTB	(70)
USA	1 Pakistan, 6N/A	1F, 6N/A	Human	Hominidae	7	1 EPTB, 6 N/A	(15)
USA	N/A	N/A	Human	Hominidae	1	N/A	(71)
USA	Southeast Asia	N/A	Cynomolgus Macaque	Cercopithecoidea	27	27 PTB & EPTB	(29)

# N/A, Not available; M, Male; F, Female.

* PTB, Pulmonary TB; EPTB, Extra pulmonary TB.

a Country of corresponding author’s institution.

Approximately 66% of reported *M. orygis* infections, whether in animals or humans, were diagnosed outside the TB endemic areas of South Asia. Of the 250 reported cases, 150 were reported in High-Income Countries (HIC) that are described traditionally as low-burden TB countries [<10/100,000 cases of TB] (23). Among the low-burden countries where *M. orygis* infections were reported, Canada, New Zealand, and the United Kingdom stood out, and interestingly, they all have endemic reservoirs of bovine tuberculosis. Concerningly, Canada and the USA, two of the most affected high-income countries, have recognized wild host species for *M. orygis*, including members of the Bovidae family (such as buffalo) and deer (24). Although low numbers of bovine TB have been reported in the USA and the Netherlands (<0.5% of total TB cases) (24, 25), and complete eradication of this disease has been achieved in Australia and Norway (24), the potential threat *M. orygis* poses to cattle populations within these countries cannot be dismissed. Furthermore, these unexpected cases have drawn significant attention from clinicians and epidemiologists. For instance, in 2012, a dairy worker from India apparently transmitted *M. orygis* to a cow in New Zealand, a country with a low incidence of TB (26). Similarly, in New York, USA, a female patient initially from South Asia developed lymph node TB secondary to an *M. orygis* infection (15). Such cases underscore the complex dynamics of *M. orygis* transmission, highlighting the need to track the pathogen’s ability to cross geographic and species boundaries.

**Figure 3:**
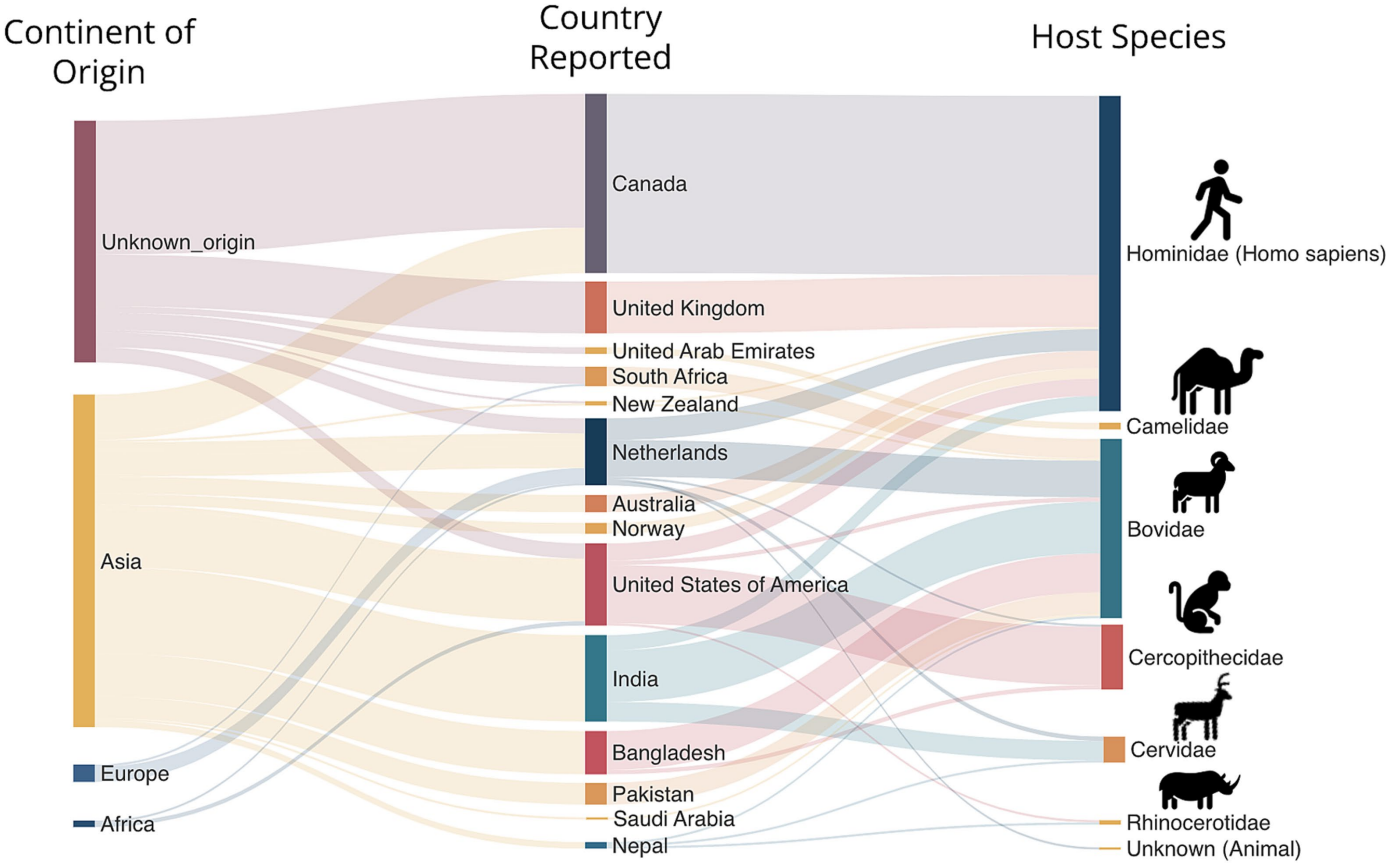
Host and geographical distribution of reported *M. orygis* cases.

Although global context on the prevalence of *M. orygis* remained understudied, certain geographical regions exhibit notably higher case densities, particularly in South Asia (Figures 2, 3) (27). For example, Duffy et al. showed that of 940 mycobacterial cultures collected between 2018 and 2019 in India the prevalence of *M. orygis* was 0.7%, surpassing *M. bovis* at 0.5%. Cultures identified as MTBC members other than *M. tuberculosis*, or those with inconclusive PCR results, were then further analyzed using whole-genome sequencing (WGS) to ensure accurate identification (28). This study provided a pivotal argument that zTB should be redefined to include other MTBC members and not just exclusively limited to *M. bovis*.

This regional concentration of *M. orygis* infections in Asia contrasts sharply with data from countries like the United States and Canada, where reported cases of *M. orygis* remain low. In the United States, approximately 9,000 cases of MTBC infection are reported annually, with the vast majority attributed to *M. tuberculosis*. However, between 2005 and 2017, data from New York State indicated a minimal incidence of *M. orygis*, though this likely does not provide a comprehensive representation of the national prevalence (29). The true prevalence is unknown, as these cases were sourced exclusively from New York State’s Health Department, potentially overlooking cases from the animal or agriculture sector.

Importation of infected animals into low TB incidence countries has been also a potential route for geographical spread highlighting the importance of quarantine measures to protect humans from potential exposure of zTB (29). Crossing of geographical borders, be it human movement via migration or import of animals, from countries with a high incidence of TB could be a plausible explanation for the of global spread of zTB caused by *M. orygis*. In Canada, a significant proportion of *M. orygis* cases (>70%) have been linked to individuals with South Asian origins. One study reported that all detected infections (21/21) were associated with this region (30). This trend aligns with the country’s diverse population and underscores the importance of understanding geographic and demographic patterns in *M. orygis* transmission, with approximately 57 cases reported in the last decade (8). Concerns also arise regarding zoonotic transmission, especially given the endemicity of known host species of *M. orygis* within Canada. In the USA, all reported macaque infections were linked to South Asian imports, and the nine human cases primarily originated in South Asia. This influx highlights the need for improved surveillance and detection measures. Canada’s experience with *M. orygis* disease has evolved notably since the first reported case in 1997. While only four cases were documented between 2007 and 2010, there has been a substantial rise, with 57 cases reported over the past decade (8). Similarly, the United Kingdom (UK), where all MTBC isolates responsible for human are routinely sequenced, has reported only 25 clinical isolates to date, suggesting a low prevalence or challenges with culture-confirmation of zTB in humans (31).

Despite significant advancements in identifying and reporting *M. orygis* cases, the true burden of this pathogen remains underrepresented due to diagnostic challenges and underreporting. The trend may suggest both progress and ongoing challenges in global TB control efforts, exacerbated by the aftermath of the SARS-CoV-2 pandemic. The spread of *M. orygis* highlights the critical need for continuous vigilance and the refinement of surveillance strategies to accurately assess the burden of this pathogen and mitigate its spread effectively.

## Affected hosts and clinical manifestations of *Mycobacterium orygis* disease

**Figure 4:**
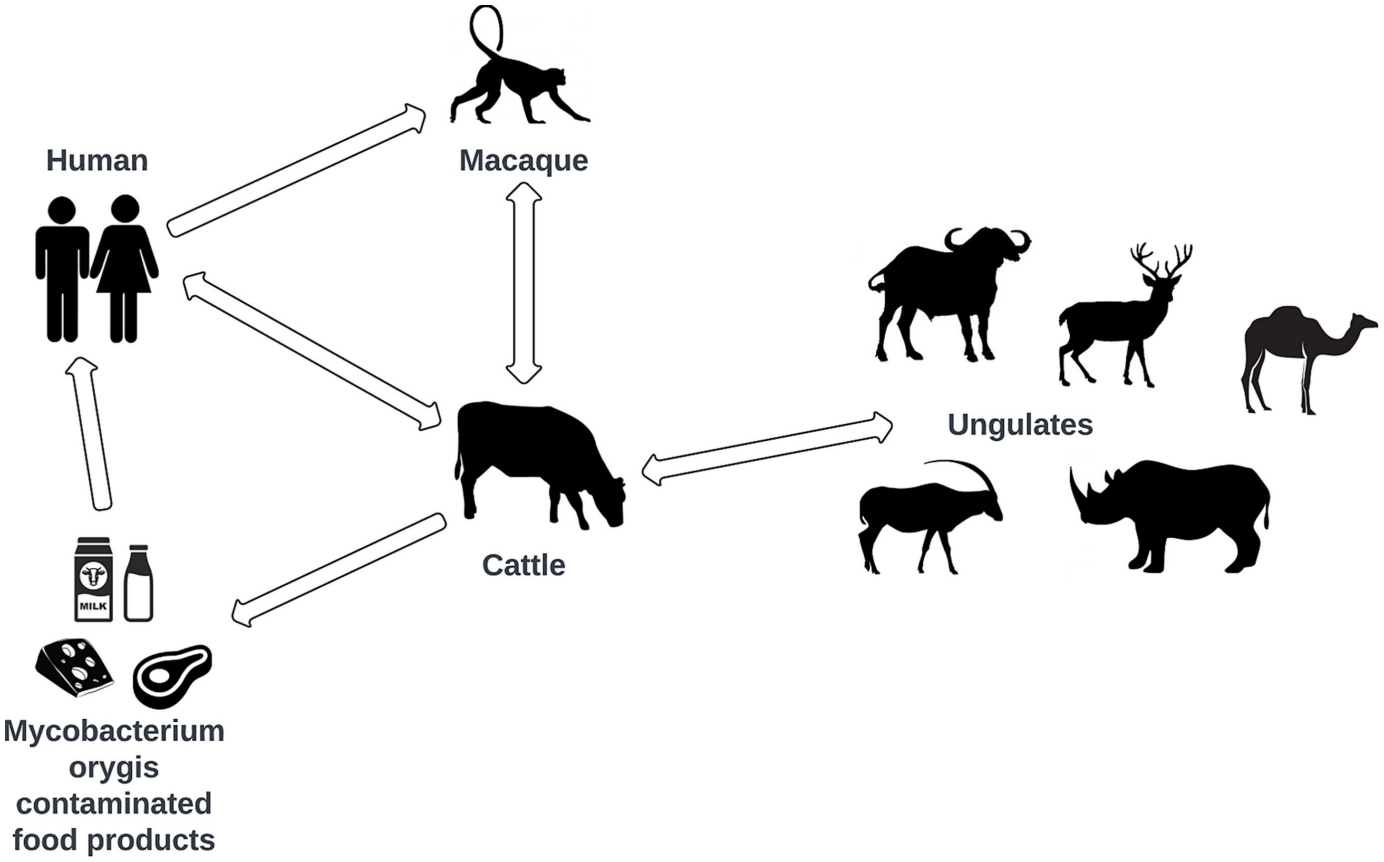
Flowchart of reported transmission chains between the various host species of *M. orygis*.

From a total of 250 cases reported to date, 67 were diagnosed in cattle, 56 in ‘other mammalian hosts’ and 127 in humans, with an apparent disparity in host infections (Figures 3, 4). Instances of human infection occurred the most compared to any other host species, with humans having the highest relative frequency, accounting for half (51.2%) of reported cases. While the frequency is high for humans, it is difficult to rule out a sampling bias due to more active investigation and reporting of zTB in humans as opposed to TB in animals. Nonetheless, *M. orygis* has been isolated from the following animal hosts: Arabian Oryx, African Buffalo, Antelope, Bison, Blackbuck, Blue Bull, Buffalo, Camel, Cattle, Cynomolgus Macaque, Deer, East African Oryx, Gazelle, Greater One Horned Rhinoceros, Rhesus Monkey, Sambar, Spotted Deer, and Waterbuck (Table 1). Notably, all infections reported to date from the African continent (including animals or specimens imported into Europe that were identified subsequently) appeared to be in wild/ captured animals, specifically in the Bovidae family (17, 32, 33). Additional research is warranted to elucidate the transmission dynamics of *M*. *orygis*, from and within Africa, as current data remains limited.

Current evidence suggests that *M. orygis* infections cause typical TB-like symptoms with both latent and active TB forms documented (15, 25, 26). Of all reported cases in animals and humans with known manifestations (Table 1; 250 cases), 59% exhibited only pulmonary disease (PTB), 41% had extrapulmonary disease (EPTB), and 32% presented with both PTB and EPTB. In humans the distribution was relatively uniformed, with 52% presenting with PTB, 48% with EPTB, and 30% with both PTB and EPTB. The observation of a more significant proportion of PTB than EPTB in *M. orygis* infections is consistent with the literature concerning *M. tuberculosis* and *M. africanum* (34) but not consistent with *M. bovis* (26, 35). A potential risk for the development EPTB from *M. orygis* has been observed via the oral consumption route of *M. orygis* bacilli (36, 37).

Susceptibility to *M. orygis* infections of different gender remains poorly understood. Riopel et al. showed a higher frequency of female than male patients in human cases infected with *M. orygis* (28, 30). Of the cases to date where the gender of the host species was reported, 26.5% (27/102) were male, and 73.5% (75/102) were female The ratio becomes even more skewed upon examining only human cases, with 25.3% male (20/79) and 74.7% (59/79) female. This trend is not consistent with typical MTBC epidemiological data. A study conducted in Victoria, Australia, concluded from less than 5,000 cases that the ratio of male to female TB cases was [1.2:1] and that males generally seek diagnosis and treatment sooner (38). In 2016, a German study analyzed 5,915 cases of TB, indicating an incidence rate nationally of 7.2:100,000, but with males having an incidence more than double the female incidence [9.9:100,000 versus 4.6:100,000] (39). The reasons for the observed disparity in *M*. *orygis* infections remained unclear. While occupational and environmental exposures could play a role, particularly in regions where women have an increased contact with cattle, definitive conclusions cannot be drawn (40, 41). Further research is needed to determine whether the pattern reflected true biological susceptibility, differences in exposure or biases in infection reporting (41, 42).

Animal host-to-host transmission has been documented in the wild. Rahim et al. identified isolates of *M. orygis* from two captured rhesus monkeys and 18 dairy cows and showed the same ‘Mycobacterial interspersed repetitive unit-variable number tandem repeat’ [MIRU-VNTR] (43) pattern in both monkeys and 15 cattle, suggesting a possible spread of this strain within the area (19). Similarly, Lipworth et al. identified 24 isolates within the United Kingdom, identifying two clusters of probable human-to-human transmission, with one genomic cluster having zero single nucleotide polymorphism (SNP) difference amongst the genomes, and the other cluster having members with up to 6 SNP difference (31). One of the most notable transmission cases was the reverse zoonotic case in New Zealand observed by Dawson et al. (26). Even though this is a rare occurrence, it is not impossible, and clinically consistent with disease caused by *M. tuberculosis* that has been observed to occur as a reverse zoonotic transmission (44). The occurrence of reverse zoonosis (zooanthroponosis) (26) (Figure 4), challenges current definition of zTB. While the definition of zTB has been proposed to include all members of the MTBC, the bidirectional flow of mycobacterial infections between humans and animals emphasizes the need for a more inclusive and nuanced definition of zTB.

## Clinical treatment strategies

There is limited evidence on drug resistance in *M*. *orygis* in the literature. Sumanth et al. identified resistance-associated genes in *M. orygis* isolates in 3 out of 8 patients in southern India. One patient was infected by a *M. orygis* strain which had a frameshift in *gid* gene, suggesting resistance to streptomycin while possible hetero-resistance was detected in the remaining two (i.e., proportion of sequencing reads with resistance conferring mutations present in *rrs, rplC* and *embB* in one of the cases, *gyrB* gene in the other) (45). All isolates, however, appeared phenotypically susceptible to first line anti-tuberculous agents.

Treatment of *M*. *orygis* follows a standard MTBC treatment regime; first line anti-tuberculous drugs (rifampicin, isoniazid, pyrazinamide and ethambutol) are employed for disease caused by wild-type susceptible strains. Treatment could be extended for the disseminated disease when necessary, or second line anti-tuberculous agents used for more resistant strains (17, 45).

## Current molecular diagnostic techniques for the detection of *Mycobacterium orygis*

Differentiating strains within the MTBC, including *M. orygis*, has historically been a challenge, necessitating the development of specialized molecular diagnostic assays as phenotypic presentation and morphology of *M. orygis* is typical of MTBC members. An early differentiation within MTBC was performed using restriction fragment length polymorphism (RFLP) typing within IS*6110* (16). However, this method has limited resolution and require a large amount of target DNA (46). MIRU-VNTR typing has also been critical in distinguishing *M. orygis* within the MTBC by identifying unique tandem repeat loci, such as the undetectable 2163b loci, which are characteristic of *M. orygis* strains (30). This method has effectively identified strain clusters in wildlife and livestock, highlighting distinct transmission dynamics and host adaptations (21, 47).

While members of MTBC are highly genetically related, each species carry insertions/deletions known as region of differences (RD) (17). RD analysis was long considered the ‘gold standard’ for MTBC identification and is still used in many centers. Similar to other MTBC species, such as *M. caprae* and *M. bovis, M. orygis* lacks specific regions, RD7, RD8, RD9, RD10, and unique regions RD^cap^, RD^bovis^ and RD^oryx1^. Moreover, *M. orygis* has preserved regions of difference, including RD1, RD2, RD4, RD5a, RD6, RD13, and N-RD25^bovis/caprae^. The presence of RD12 in ‘oryx bacillus’ was reported in one earlier paper, although Van Ingen et al. and Rahim et al. found a deletion (17, 19). Clarification via NCBI and the European Nucleotide Archive confirmed the deletion of RD12 but affirmed the presence of RD1, RD2, RD4, RD5a, RD6, and RD13 in *M. orygis*. All major MTBC RD differences have been laid out (Table 2). Importantly, there are more specific regions within *M. orygis* genome which can be targeted for further speciation such as RD2seal, RD12oryx and RDcap_Spain1 (9), among others. RD analysis can also be used in conjunction with SNPs to clarify speciation (9, 48, 49).

**Table 2:** Presence/absence matrix of ROD for members of the MTBC complex.

MTBC species	RD1	RD2	RD4	RD5	RD7	RD8	RD9	RD10	RD12	RD13
*M. canettii*	+	+	+	+	+	+	+	+	+	+
*M. tuberculosis*	+	+	+	+	+	+	+	+	+	+
*M. africanum*	+	+	+	+	+	+	−	+	+	+
*M. mungi*	+	+	+	+	−	−	−	−	+	+
*M. suricattae*	+	+	+	+	−	−	−	−	+	+
*M. microti*	−	+	+	+	−	−	−	−	+	+
*M. pinnipedii*	+	+	+	+	−	−	−	−	+	+
*M. orygis*	+	+	+	+	−	−	−	−	+	+
*M. caprae*	+	+	+	−	−	−	−	−	−	−
*M. bovis*	+	+	−	−	−	−	−	−	−	−
*M. bovis BCG*	−	−	−	−	−	−	−	−	−	−

[[(+) is positive for presence, (−) is Negative for deletion].[(+) is positive for presence, (−) is Negative for deletion].[(+) is positive for presence, (−) is Negative for deletion].[(+) is positive for presence, (−) is Negative for deletion].[(+) is positive for presence, (−) is Negative for deletion].[(+) is positive for presence, (−) is Negative for deletion].[(+) is positive for presence, (−) is Negative for deletion].[(+) is positive for presence, (−) is Negative for deletion].[(+) is positive for presence, (−) is Negative for deletion].[(+) is positive for presence, (−) is Negative for deletion].[(+) is positive for presence, (−) is Negative for deletion].[(+) is positive for presence, (−) is Negative for deletion].[(+) is positive for presence, (−) is Negative for deletion].[(+) is positive for presence, (−) is Negative for deletion].[(+) is positive for presence, (−) is Negative for deletion].[(+) is positive for presence, (−) is Negative for deletion].[(+) is positive for presence, (−) is Negative for deletion].[(+) is positive for presence, (−) is Negative for deletion].[(+) is positive for presence, (−) is Negative for deletion].[(+) is positive for presence, (−) is Negative for deletion].[(+) is positive for presence, (−) is Negative for deletion].[(+) is positive for presence, (−) is Negative for deletion].[(+) is positive for presence, (−) is Negative for deletion].[(+) is positive for presence, (−) is Negative for deletion].[(+) is positive for presence, (−) is Negative for deletion].[(+) is positive for presence, (−) is Negative for deletion].[(+) is positive for presence, (−) is Negative for deletion].[(+) is positive for presence, (−) is Negative for deletion].[(+) is positive for presence, (−) is Negative for deletion].].

## Genomic diversity of *Mycobacterium orygis*

**Figure 5:**
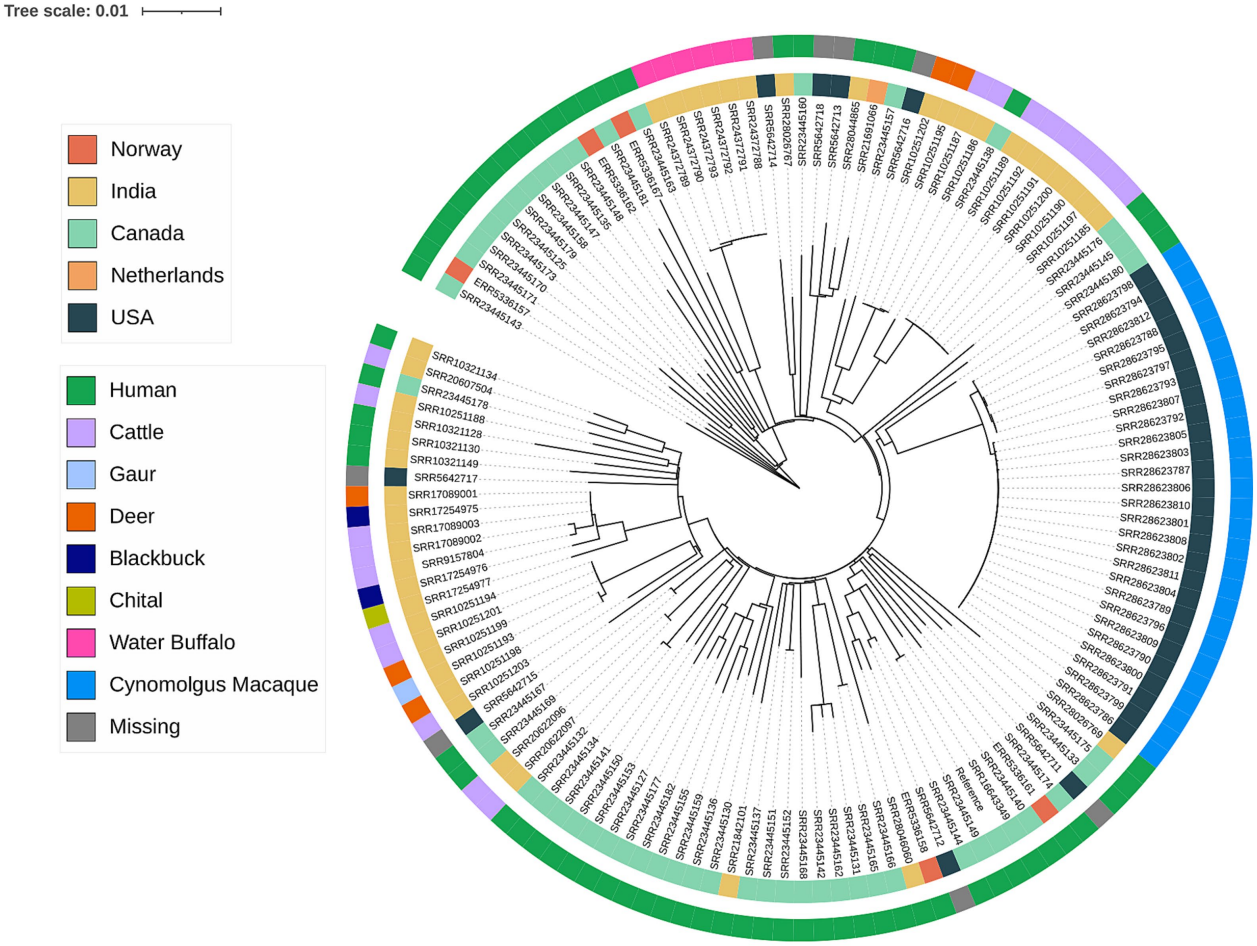
Phylogenetic tree of available *M. orygis* genomes. Sequencing reads or contigs were aligned to *M. orygis* strain 51,145 (GenBank accession CP063804) as the reference. Genomes that aligned to less than 90% of the reference were omitted. A maximum-likelihood tree was inferred from the whole genome SNP alignment using IQ-TREE version 2.3.6 using the GTR + F + R2 model with 1,000 replicates for ultrafast bootstrap. Phylogenetic tree was generated using I-TOL (https://itol.embl.d).

Whole Genome Sequencing (WGS) provides the ultimate high-resolution identification of MTBC species and insights into the phylogeny and the evolution within the MTBC. As of Nov 2024, there were 153 *M. orygis* genomic sequences uploaded onto public repositories. Inferences into the phylogeny of *M. orygis* revealed genomic diversity with pairwise SNP differences ranging from 0 to 523 core SNPS. Interestingly, genomes that clustered together (0 SNPs) did not always show stratification by host, suggesting a complex transmission dynamic that warrants further investigation (Figure 5). The analysis of virulence genes has pinpointed crucial loci within the ESX-1 secretion system, including the ESAT-6 and CFP-10 genes, which contribute to pathogenicity in *M. orygis* (50), namely, disruption of host cell membranes and regulation of host immune response (51, 52).

The continuing advancement of sequencing technologies and accessibility of WGS have aided our understanding of zTB, paving the way for faster diagnostics and genomic surveillance. Targeted metagenomics was recently endorsed by the WHO as a method for rapid and accurate TB diagnosis (53). This style of sequencing focuses on specific genomic regions, such as 16S rRNA or pathogen-specific SNPs, to provide precise insights into microbial diversity and adaptations in less time than conventional culture-based methods. *M. orygis*-specific SNPs incorporated into targeted metagenomics offers a high-resolution diagnostic tool for its identification and characterization (54). Additionally, adapting RD-based SNPs into a PCR panel could further improve targeted diagnostics in the short term, enabling accurate detection and analysis of genetic variations.

## Conclusion

In conclusion, there is increasing evidence of *M. orygis* emergence and spread as a human and zoonotic pathogen, particularly in South Asia. The understanding of the organism’s transmission and magnitude of its emergence is complicated by the complexities of tuberculosis epidemiology and the challenges in accurate diagnosis and reporting. The higher frequency of female patients in *M. orygis* infections, in contrast to *M. tuberculosis*, suggests possible distinct risk factors, highlighting the need for further research into the epidemiology of the disease. Furthermore, there are concerns that *M. orygis* may be circulating in wild animal and livestock populations with significant implications for the transmission and spread of the disease within domesticated and food producing animals. To address these challenges, it is essential to prioritize investment into enhance laboratory diagnosis, surveillance strategies for zTB and epidemiological studies. Strengthening these efforts will be key in controlling the spread of zTB and alleviating its impact on both human and animal populations globally.
